# Evaluation of surface changes of dental implants after irradiation with diode laser beams with different energies: A SEM study

**DOI:** 10.15171/japid.2018.013

**Published:** 2018-12-25

**Authors:** Masoumeh Faramarzi, Mehrnoosh Sadighi, Sayeh Mirhashemi

**Affiliations:** ^1^Department of Periodontics and Dental and Periodontal Research Center, Faculty of Dentistry, Tabriz University of Medical Sciences, Tabriz, Iran; ^2^Dentist, Private Practice, Tehran, Iran

**Keywords:** Diode laser, Iimplant, scanning electron microscopy

## Abstract

**Background:**

The aim of the current study was to evaluate implant surface changes following radiation with diode laser beams at various energy levels.

**Methods:**

Twenty implants (Dentis, Korea) were irradiated with diode laser, and two implants were considered as controls. The samples were irradiated at energies of 1.5, 2.5, 3.5, 4.5, 5.5 W for 5 and 10 seconds. Then surface implant changes were evaluated using Scanning Electron Microscopy (SEM).

**Results:**

At irradiation with laser beam energies of 1.5, 2.5, 3.5 W, there were no significant morphologic changes and any melting on implants and the surfaces in SEM analyses were similar to the control group surfaces. However, irradiation with 4.5 and 5.5 W for 5 and 10 seconds resulted in surface changes. In particular, after irradiation with 5.5-W diode laser beams for 10 seconds, extensive melting was visible.

**Conclusion:**

The results of the current study showed that diode laser beams up to 3.5 W did not damage implant surfaces; therefore, they might be useful for treatment of peri-implantitis.

## Introduction

In recent years, dental implants have been used as a common treatment.^1^ If the implant surface is colonized with bacteria, the tissues around the implant will be affected. Peri-implantitis is described as an inflammatory disease of the peri-implant tissues that results in bone loss.^2^

In mucositis the inflammation is limited to the mucosa and the bone around the implant is not affected.^2^ According to the literature, the peri-implantitis microbiota is very similar to advanced periodontitis.^3^ In order to determine the real risk factors of diseases around implants, prospective studies of peri-implant diseases should be carried out, and to date limited studies have been published. it was proven that inappropriate oral hygiene, previous periodontitis and smoking are risk indicators for peri-implantitis. It is not surprising that peri-implantitis therapy is based on the proposed periodontitis treatment aimed at reducing bacterial loading of the pockets around the implants.^4^ Therapeutic modalities for peri-implantitis include mechanical debridement and chemical treatment.^5,6^ Mechanical debridement can cause changes in implant surfaces, preventing bone regeneration. Inadequate debridement of bacterial colonization and endotoxins will lead to disease relapse. Chemical surface treatment is not applicable to all the implant surfaces and also it has limited effects on the removal of the plaque.^7,8^ Recent studies have shown that lasers are useful in implant surface decontamination. Lasers that are often used to treat diseases around implants include diode, CO_2_ and Erbium lasers, due to the hemostatic properties, selective calculus removal and bactericidal effects.^9-12^ Diode lasers with a wavelength of 980 or 810 nm are the most commonly used lasers. The target of these lasers is pigments in soft tissue such as melanin and hemoglobin. Higher wavelengths are better absorbed in water. Therefore, 980-nm diode laser is safer and more useful around the implant.^13^ Romanos, in a study, suggested that a 960-nm wavelength diode laser, even in higher power settings, is safe to use in titanium implants, but 810-nm wavelength might damage implant surfaces.^14^ Therefore, 980-nm diode is believed to be the only useful laser in the treatment of implants, but with some limitations in deep and efficiency of cutting. Its main advantage is small size and relatively low cost.^12,13^ The aim of the present investigation was to analyze the possible morphological alterations of the implant surfaces after application of diode laser irradiation at various times and energies.

## Methods

A total of 22 implants (Dentis® Dental Implants, Daegu, Korea) with RBM (Resorbable Blasted Media) surface served as substrates. The implants were mounted in stone. While the upper three threads were exposed. The surface roughness was Ra = 1.5±0.2 µm according to the manufacture. The samples were divided into 5 groups (n=5) while 2 implants served as controls.

The groups were irradiated with the following output powers: 1.5, 2.5, 3.5, 4.5 and 5.5 W, respectively.

In each group two samples were irradiated for 5 seconds and the other two were irradiated for 10 seconds. A diode laser with the wavelength of 810 nm with a 600-µ fiber in contact mode were used for the study. Surface implant effects were examined under a scanning electron microscope (SEM). All the experiments were carried out on the same day. The preparations for SEM analysis were performed on the day after, and photos were taken by SEM. Each implant surface was evaluated for changes in morphology, melting and surface alterations.

### Scanning Electron Microscopy

Implants from each group were placed in a scanning electron microscope vacuum chamber (CamScan MV2300, Electron Optic Services Inc., Ottawa, Canada) and microphotographs were taken at different magnifications (×80, ×300, ×500, ×1500 and × 3000) in order to assess the surface topography.

## Results

**Figure 1:**
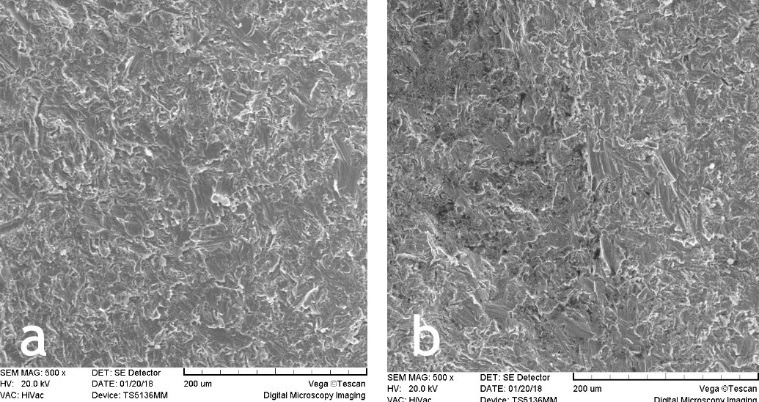
Scanning electron eicroscope (SEM) images at 1.5 W; 5 (a) and 10 (b) seconds (original magnification ×500).

**Figure 2:**
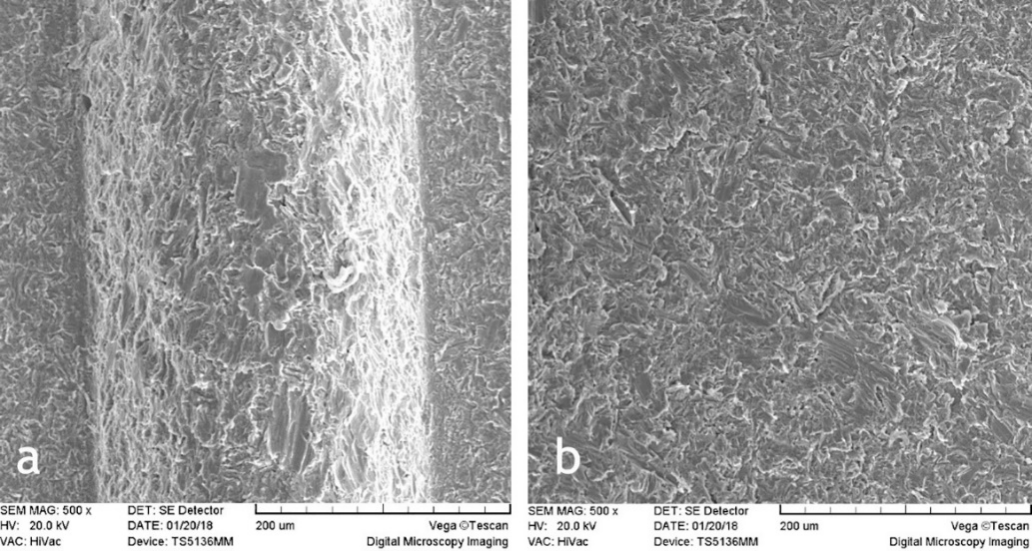
Scanning electron microscopy (SEM) images at 2.5 W; 5 (a) and 10 (b) seconds (original magnification** ×**500).

**Figure 3:**
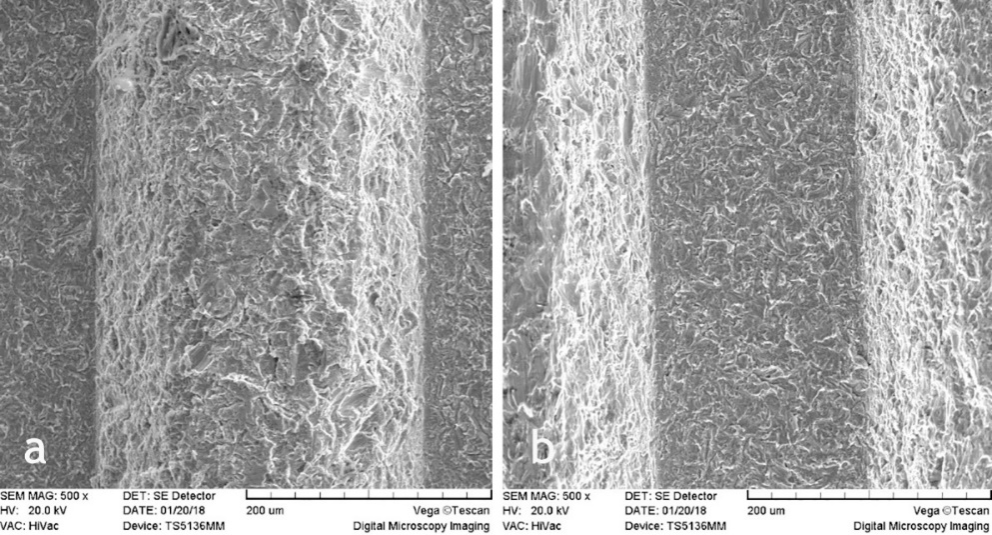
Scanning electron microscope (SEM) images at 3.5 W; 5 (a) and 10 (b) seconds (original magnification ×500).

**Figure 4:**
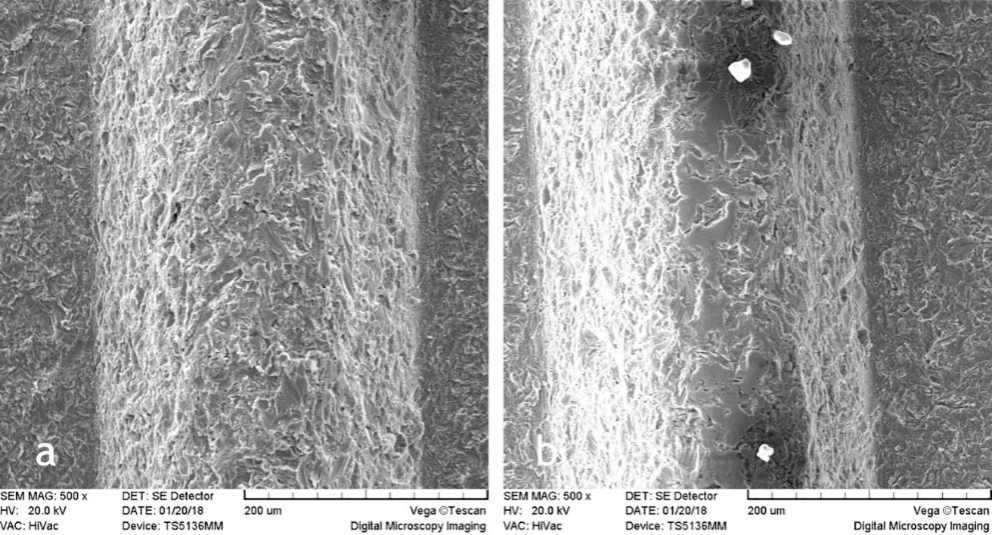
Scanning electron microscope (SEM) images at 4.5 W; 5 (a) and 10 (b) seconds (original magnification ×500).

**Figure 5:**
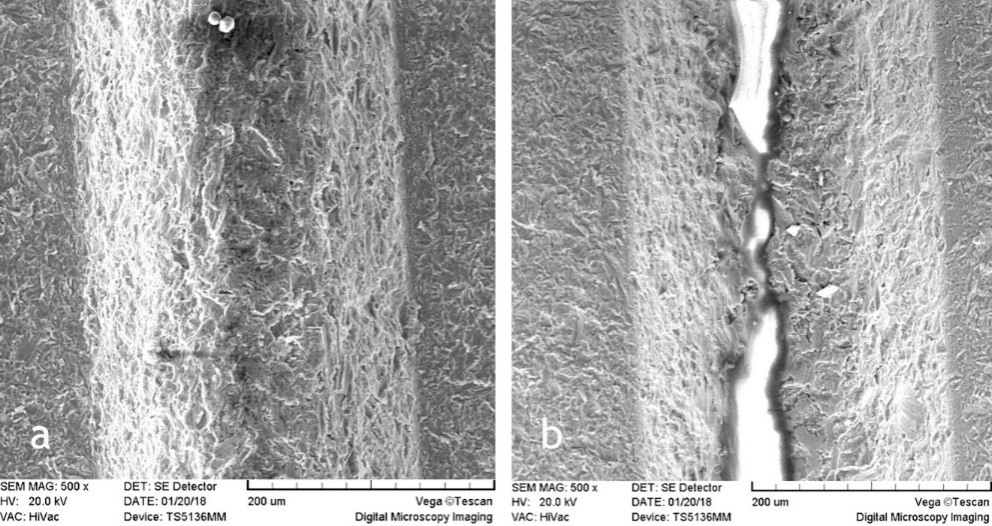
Scanning electron microscope (SEM) images at 5.5 W; 5 (a) and 10 (b) seconds (original magnification ×500).

In this study, 22 implants were evaluated for surface changes induced by diode laser beams. Surface changes were different depending on energy settings. The experiments were carried out at energies of 1.5, 2.5, 3.5, 4.5, 5.5 W for 5 and 10 seconds. SEM evaluations did not show surface damage in any of the titanium implants that were irradiated with 1.5, 2.5 and 3.5 W for 5 and 10 seconds (Figures 1‒3). Melting and surface damage was reported at energies of 4.5 and 5.5 W for 5 and 10 seconds (Figures 4 and 5). These results suggested that diode laser up to 3.5 watts is useful for decontamination and debridement of implant surfaces in cases of peri-implantitis.

## Discussion

Replacement of teeth with implants is a predictable therapy in the majority of patients. If the surface of the implant is colonized by pathogenic bacteria, the plaque-related inflammation results in tissue destruction around the implant or peri-implantitis.^14^ Different methods have been proposed for the treatment of peri-implantitis and disinfection of the implant surfaces. In addition to conventional (mechanical and chemical) therapies, various lasers have also been used to treat peri-implantitis.^15^ Contemporary researches have suggested that mechanical debridement of the surface of the implant by scalers has an unfavorable effect on the implant surfaces and causes implant surface roughness. Plastic curettes also take bacteria ineffectively.^16,17^ The advantage of lasers is that it is a painless process and creates excellent homeostasis; therefore, a blood-free background is created, with faster wound healing and

less anesthesia requirement.^18^ Eriksson showed that a temperature of 47ºC for one minute caused irreversible bone destruction; therefore, it is crucial that the parameters of the lasers do not exceed this biological temperature.^19^ The laser used in this study was a diode laser that examined changes in implant surfaces at energies of 1.5, 2.5, 3.5, 4.5 and 5.5 W for 5 and 10 seconds. In this research, based on our findings, the first hypothesis was rejected, stating that the effect of different levels of diode laser energy on dental implant surfaces is the same as that of the control group. The purpose of the treatment of peri-implantitis is to remove bacterial contamination from the surface of implants without any changes in titanium surfaces, as any changes in implant surface can disturb osseointegration, leading to loss of the implant. Therefore, use of a high-power diode laser leads to superficial changes. In a study by Stefan Stubinger to investigate the surface changes induced by diode laser radiation (1-3 W) for 10 seconds in continuous wave mode on the SLA surface implant, no changes in implant surfaces were observed.^20^ The results of this study are consistent with our study. A study by Romanos et al. on the diode laser effects on titanium disks with 5, 10 and 15 W and 980-nm diode laser did not show any significant changes in implant surfaces.^21^ This finding is different from the results of our study, which can be attributed to the difference in wave length of diode laser used.

However, the results of the current research showed that with diode laser use, up to 3.5 W, the surface morphology of the laser-irritated implant was similar to the untreated implants. The SEM images indicated that the clinical application of diode lasers in cases of peri-implantitis had no risk or unfavorable effect on the implants.

## Authors’ contributions

The study was planned by MF and MS. Data collection was carried out by SM; statistical analyses and interpretation of data were carried out by MS. The manuscript was prepared by MS and edited by MF. All the authors have read and approved the final manuscript for submission.

## Competing interests

The authors declare no conflict of interests

## Ethics approval

We certify this article does not contain any studies with human participants or animals performed by any of the authors.
